# Annexin A5 reduces infarct size and improves cardiac function after myocardial ischemia-reperfusion injury by suppression of the cardiac inflammatory response

**DOI:** 10.1038/s41598-018-25143-y

**Published:** 2018-04-30

**Authors:** Rob C. M. de Jong, Niek J. Pluijmert, Margreet R. de Vries, Knut Pettersson, Douwe E. Atsma, J. Wouter Jukema, Paul H. A. Quax

**Affiliations:** 1 Department of Surgery, Leiden University Medical Center, Albinusdreef 2, P.O. Box 9600, 2300 RC Leiden, The Netherlands; 2 Einthoven Laboratory for Experimental Vascular Medicine, Leiden University Medical Center, Albinusdreef 2, P.O. Box 9600, 2300 RC Leiden, The Netherlands; 3 Department of Cardiology, Leiden University Medical Center, Albinusdreef 2, P.O. Box 9600, 2300 RC Leiden, The Netherlands; 4 Athera Biotechnologies, Mölndal, Sweden

## Abstract

Annexin A5 (AnxA5) is known to have anti-inflammatory and anti-apoptotic properties. Inflammation and apoptosis are key processes in post-ischemic cardiac remodeling. In this study, we investigated the effect of AnxA5 on left ventricular (LV) function and remodeling three weeks after myocardial ischemia-reperfusion (MI-R) injury in hypercholesterolemic ApoE*3-Leiden mice. Using a mouse model for MI-R injury, we demonstrate AnxA5 treatment resulted in a 27% reduction of contrast-enhanced MRI assessed infarct size (IS). End-diastolic and end-systolic volumes were decreased by 22% and 38%, respectively. LV ejection fraction was increased by 29% in the AnxA5 group compared to vehicle. Following AnxA5 treatment LV fibrous content after three weeks was reduced by 42%, which was accompanied by an increase in LV wall thickness of the infarcted area by 17%. Two days and three weeks after MI-R injury the number of cardiac macrophages was significantly reduced in both the infarct area and border zones following AnxA5 treatment compared to vehicle treatment. Finally, we found that AnxA5 stimulation leads to a reduction of IL-6 production in bone-marrow derived macrophages *in vitro*. AnxA5 treatment attenuates the post-ischemic inflammatory response and ameliorates LV remodeling which improves cardiac function three weeks after MI-R injury in hypercholesterolemic ApoE*3-Leiden mice.

## Introduction

Acute myocardial infarction (MI) initiates a massive inflammatory response^1,2^ and cell death^3^. To limit myocardial damage due to these processes and salvage ischemic myocardium, primary percutaneous coronary intervention is the preferred clinical therapy to achieve reperfusion^4^. However, post-ischemic reperfusion itself causes reperfusion injury with the formation of reactive oxygen species which cause direct cell death and stimulation of signal transduction to generate inflammatory cytokines^5^. Furthermore, it has been shown that reperfusion induces and in particular aggravates apoptosis^6,7^. Through binding and ingestion of dying cells, myeloid cells can markedly influence immune responses by enhancing or suppressing inflammation indicating close interaction between cell death and inflammation^8^. In line with this affecting apoptosis and inflammation to mitigate cellular damage might result in new clinical therapies.

Myocardial ischemia-reperfusion (MI-R) induced apoptosis results in different (intra)cellular changes including loss of the asymmetric distribution of plasma membrane phospholipids. Normally, the choline-containing lipid phosphatidylcholine is present on both the outer and inner membrane leaflet while aminophospholipids, like phosphatidylserine (PS), are concentrated on the inner membrane leaflet of viable cells. During early apoptosis and inflammatory cell activation, PS is externalized to the outer cell surface as a result of the activated proteolytic enzyme caspase-3, where it functions as an “eat me” signal to ensure early recognition and phagocytosis^9,10^. Annexins are a family of phospholipid-binding proteins and in particular annexin A5 (AnxA5) binds reversibly, specifically and with high affinity to PS-expressing cells^11^. In addition to the first discovered anti-thrombotic effects of AnxA5^12,13^, it is also known to have possible diagnostic properties in visualizing cell death^14^ including assessment of atherosclerotic plaque vulnerability^15^.

MI is reported to cause increased endogenous AnxA5 plasma levels^16^ and uptake in the infarct area^17^ in patients. After an ischemic event, cardiomyocytes were found to express PS on their cell surface for at least 6 hours. Administration of exogenous AnxA5 resulted in cytoplasmic internalization and restored sarcolemmal PS asymmetry with no externalized PS remaining, thereby possibly reversing the apoptotic process^18^. Furthermore, a reduced post-interventional inflammatory response was observed following AnxA5 treatment resulting in a potential therapeutic effect against post-interventional intimal hyperplasia^19^ and accelerated atherosclerosis^20^.

Taken together, the anti-apoptotic and anti-inflammatory effects of AnxA5, can provide a possible role of human recombinant AnxA5 as a therapeutic agent to decrease post-ischemic left ventricular (LV) remodeling and improve cardiac function. A previous study showed beneficial effects of Diannexin, a dimer of AnxA5, treatment in rabbits on post-ischemic blood flow following ischemia and reperfusion^21^. In the current study we demonstrate the beneficial effects of AnxA5 treatment on infarct size (IS) and post-infarctional cardiac remodeling in a clinically more relevant setting, namely by starting the treatment post-reperfusion and using a follow-up up to three weeks. Cardiac function and IS were assessed three weeks post reperfusion. Moreover, the experiments are performed under hypercholesterolemic conditions by using ApoE*3-Leiden mice on a Western-type diet. Furthermore, we investigate the effect of AnxA5 treatment on the post ischemic-reperfused inflammatory response.

## Results

### Annexin A5 accumulates in the infarct area

**Figure 1:**
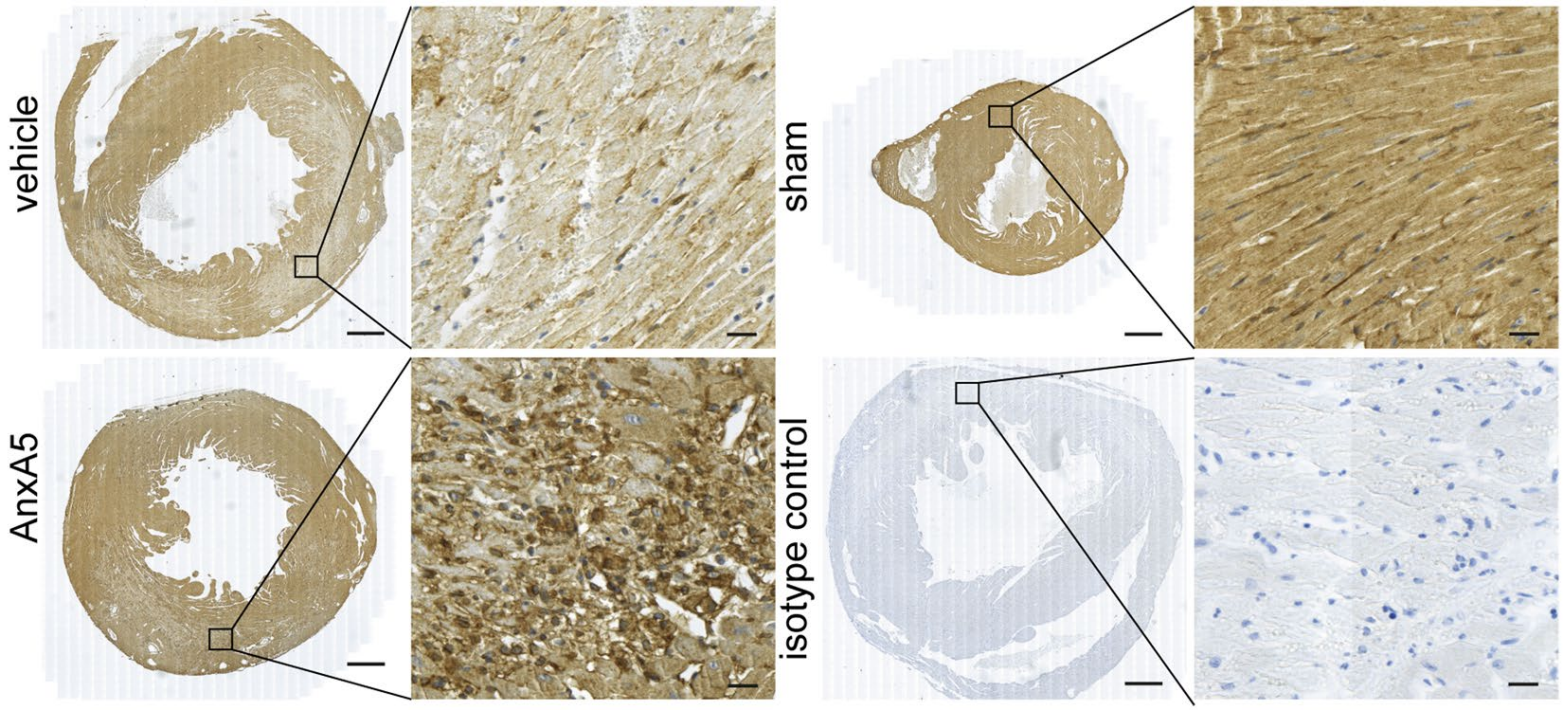
Annexin A5 staining. Representative images of cross-sections from the whole heart (left) or the infarct area (right) two days after MI-R stained for human annexin A5 using a specific AnxA5 antibody, while counterstaining was performed using haematoxilin. AnxA5 treated mice showed a more intense staining compared to vehicle and sham mice. Left panel scale bar: 500 μm, right panel scale bar: 20 μm.

First, AnxA5 accumulation in the infarct area following AnxA5 treatment was evaluated using immunohistochemistry. Two days post MI-R AnxA5 staining, using a specific AnxA5 antibody, was clearly more intense in the infarct area of AnxA5 treated when compared to vehicle treated mice (Fig. 1), suggesting AnxA5 accumulation in the infarct area. Furthermore, as can be appreciated from the images of sham operated mice, AnxA5 protein is present in the healthy myocardium. However, compared the infarcted myocardium, healthy myocardium shows AnxA5 staining which is evenly distributed throughout the tissue. Moreover, the exogenously added AnxA5 accumulates especially in the infarcted area with a spot-wise pattern. Total plasma cholesterol concentration, triglyceride levels and body weight were not affected by AnxA5 treatment (Supplementary Table S1).

### Annexin A5 reduces contrast-enhanced MRI assessed LV infarct size

**Figure 2:**
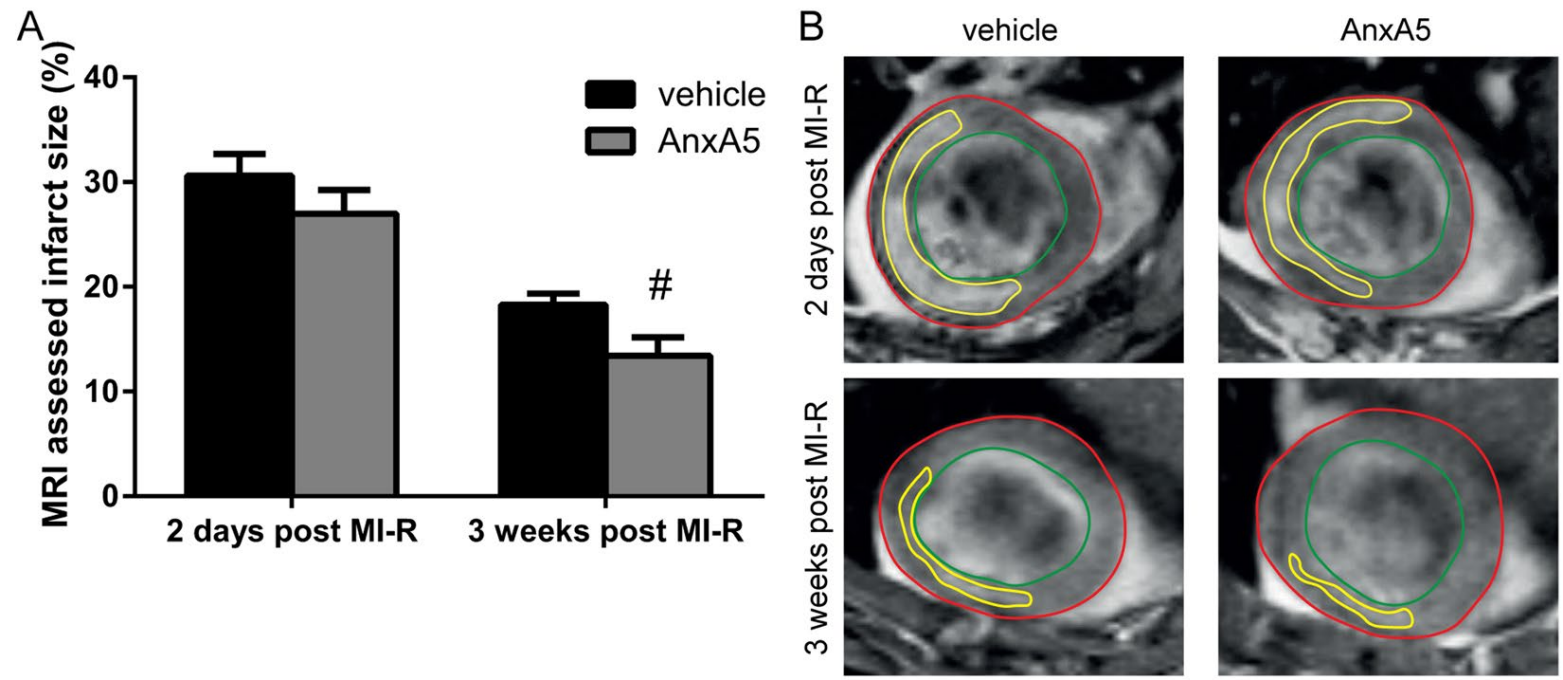
Contrast-enhanced MR imaging. Two days after MI-R no difference in infarct size was observed between the groups (n = 13–15). However, after three weeks the AnxA5 group displayed a reduced infarct size as compared to the vehicle group (**A**). Representative Gd-DPTA-enhanced MR images (**B**) two days and three weeks after MI-R of the vehicle and AnxA5 group, red line indicates epicardial border, green line indicates endocardial border and yellow line indicates infarct area. Data are mean ± SEM. ^#^P < 0.05 vs. vehicle.

Infarct size as assessed by contrast-enhanced MRI was significantly reduced after three weeks in AnxA5 treated mice compared to the vehicle group (13.4 ± 1.8% vs. 18.3 ± 1.1%, P = 0.022; Fig. 2A), while initial IS, two days after MI-R, was comparable in both groups (27.0 ± 2.3% vs. 30.6 ± 2.1%, P = 0.249; Fig. 2A). Interestingly, IS was significantly smaller (Supplementary Fig. S1A) three weeks post-reperfusion compared to two days post-reperfusion indicating infarct healing and resorption of acute infarct edema. This observation was confirmed by the absolute numbers of IS and viable myocardium. IS was decreased in both the vehicle and AnxA5 group after three weeks when compared to two days (Supplementary Fig. S1B), while viable myocardium was unchanged in time (Supplementary Fig. S1C). Reduced IS does not affect heart weight, since heart weight was comparable in all three groups (AnxA5: 145 ± 5 mg, vehicle: 140 ± 7 mg, and sham 144 ± 8 mg; Supplementary Table S1).

### Annexin A5 improves LV function

**Figure 3:**
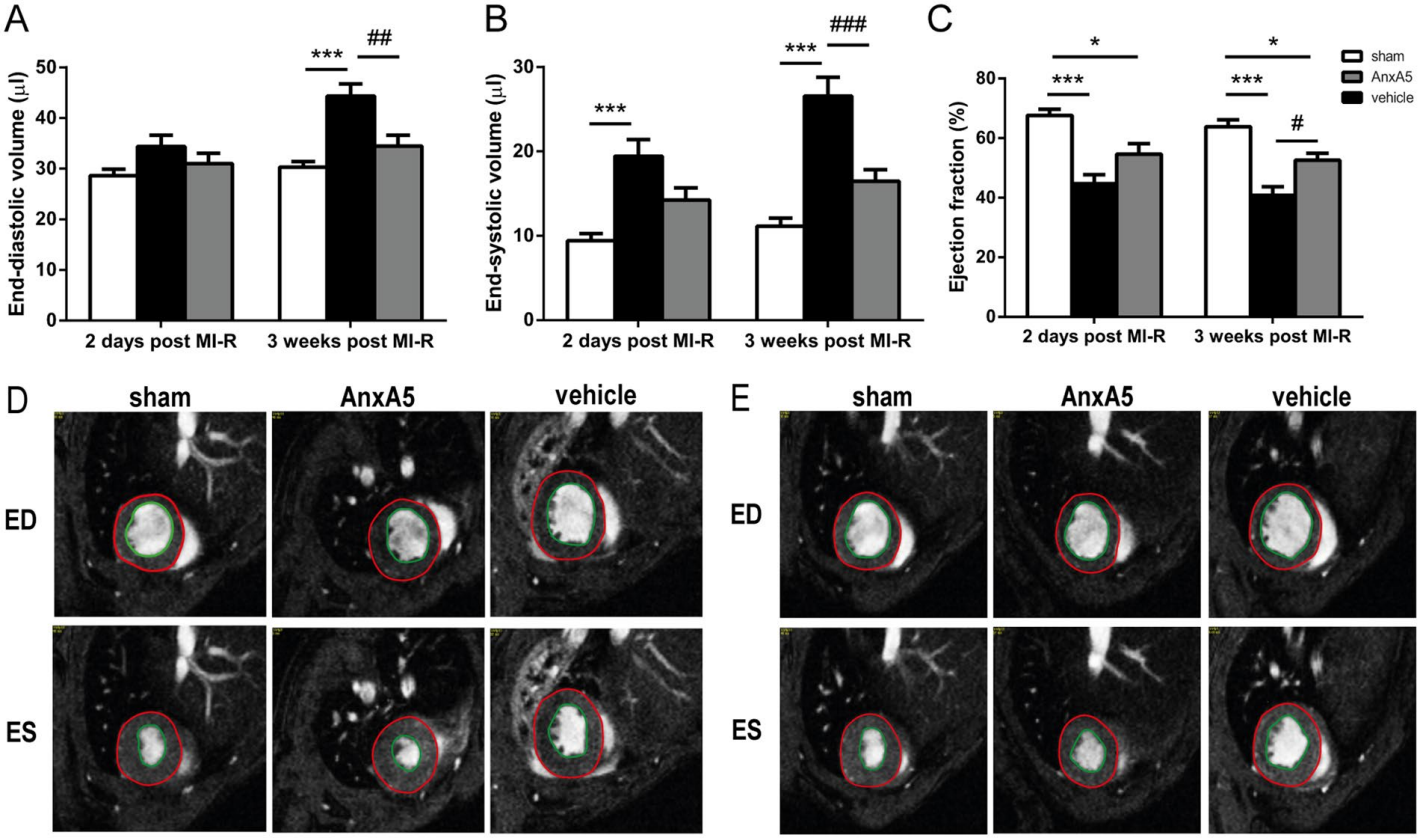
Cardiac MR imaging of LV volumes and function. Assessment of LV volumes and function two days and three weeks after MI-R (n = 12–15). AnxA5 therapy prevented the increase in end-diastolic volume (EDV) (**A**) and end-systolic volume (ESV) (**B**) accompanied by a preserved ejection fraction (EF) (**C**) as compared to vehicle three weeks post MI-R. Representative transversal short-axis MR images at end-diastole (ED) and end-systole (ES) two days (**D**) and three weeks (**E**) following MI-R in the sham, AnxA5 and vehicle groups, red line indicates epicardial border and green line indicates endocardial border. Data are mean ± SEM. ^#^P < 0.05, ^##^P < 0.01, ^###^P < 0.001 all vs. vehicle, *P < 0.05 and ***P < 0.001 both vs. sham.

MRI analyses showed that the reduced scar expansion after MI-R due to AnxA5 treatment was accompanied by a limitation of LV dilation and preserved LV function after three weeks. No differences were observed at day two (EDV: 31.0 ± 2.0 μl vs. 34.4 ± 2.3 μl, P = 1.000; Fig. 3A, and ESV: 14.2 ± 1.5 μl vs. 19.4 ± 2.0 μl, P = 0.153; Fig. 3B). However, after three weeks EDV was significantly smaller in the AnxA5 group as compared with vehicle (34.5 ± 2.2 μl vs. 44.4 ± 2.4 μl, P = 0.004; Fig. 3A). Furthermore, ESV was significantly smaller in the AnxA5 group as compared with vehicle (16.5 ± 1.4 μl vs. 26.6 ± 2.2 μl, P < 0.001; Fig. 3B).

AnxA5 seemed to have a preventive effect with respect to post-ischemic LV dilation according to nearly equal LV volumes as compared to the sham group after three weeks (EDV: 30.4 ± 1.2 μl, P = 1.000; Fig. 3A, and ESV: 11.2 ± 1.0 μl, P = 0.188; Fig. 3B), in contrast to vehicle treatment which caused obvious LV dilation (both P < 0.001; Fig. 3A,B).

The limited LV dilation was associated with a significantly better LV function, as expressed by preserved EF in the AnxA5 group compared to vehicle (52.5 ± 2.4% vs. 40.8 ± 2.9%, P = 0.019; Fig. 3C) while no significant difference was observed after two days (54.6 ± 3.5% vs. 44.7 ± 3.0%, P = 0.074; Fig. 3C).

### Annexin A5 reduces LV fibrous content and preserves LV wall thickness

**Figure 4:**
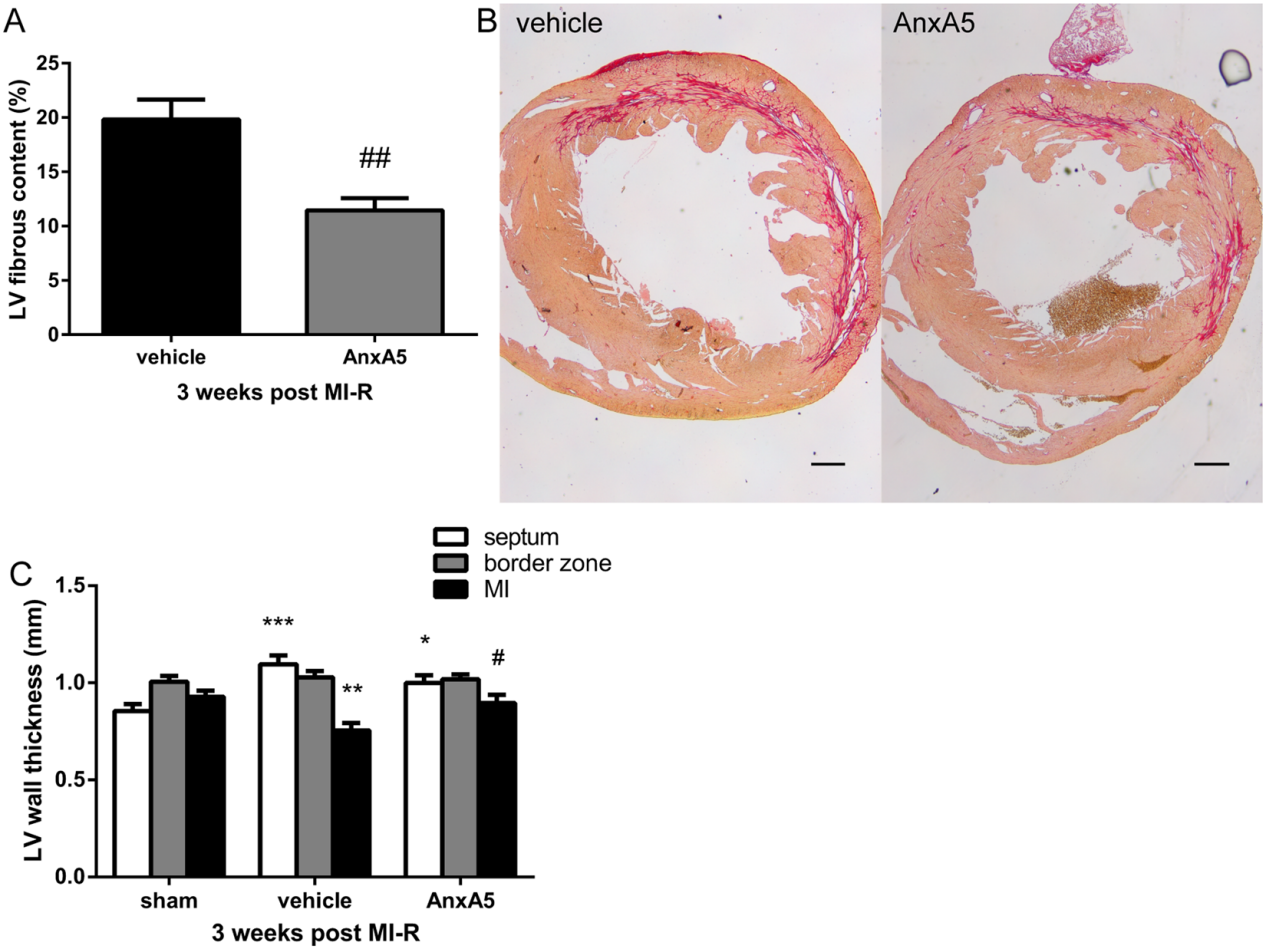
LV fibrous content and LV wall thickness. Histological analysis after three weeks (n = 9–10) showed a significant reduced LV fibrous content in the AnxA5 group compared to the vehicle group (**A**). Representative images (**B**) of Sirius red staining of cross-sections of the whole heart in the vehicle group and the AnxA5 group. Scale bar: 500 μm. LV wall thickness was significantly increased in the infarct area after AnxA5 treatment (**C**). Data are mean ± SEM. ^#^P < 0.05 vs. vehicle, *P < 0.05, **P < 0.01, ***P < 0.001 all vs. sham.

Histological evaluation of LV fibrous content endorsed the aforementioned results as assessed by cardiac MRI. AnxA5 administration caused a reduction of LV fibrous content three weeks after MI-R as compared to the vehicle group (11.4 ± 1.1% vs. 19.8 ± 1.8%; P = 0.001, Fig. 4A). This was accompanied by an increased wall thickness of the infarcted LV wall in the AnxA5 compared to the vehicle group (0.90 ± 0.04 mm vs. 0.75 ± 0.04 mm, P = 0.041; Fig. 4C). Wall thickness of the border zone area (1.02 ± 0.02 mm vs. 1.03 ± 0.03 mm, P = 1.000) and interventricular septum (1.00 ± 0.04 mm vs. 1.10 ± 0.04 mm, P = 0.326) were not affected by AnxA5 therapy as compared to vehicle (Fig. 4C).

Furthermore, LV wall thickness in the infarct area was decreased in the vehicle group compared to the sham group (0.93 ± 0.03 mm; P = 0.008), while LV wall thickness in the infarct area was preserved in the AnxA5 group (P = 1.000) when compared to the sham group (Fig. 4C). Besides, both the AnxA5 (P = 0.047) and vehicle (P = 0.001) group showed an increased wall thickness of the interventricular septum compared to the sham group (0.85 ± 0.04 mm) indicating compensatory concentric hypertrophy (Fig. 4C).

### Annexin A5 causes reduction of the local inflammatory response

**Figure 5:**
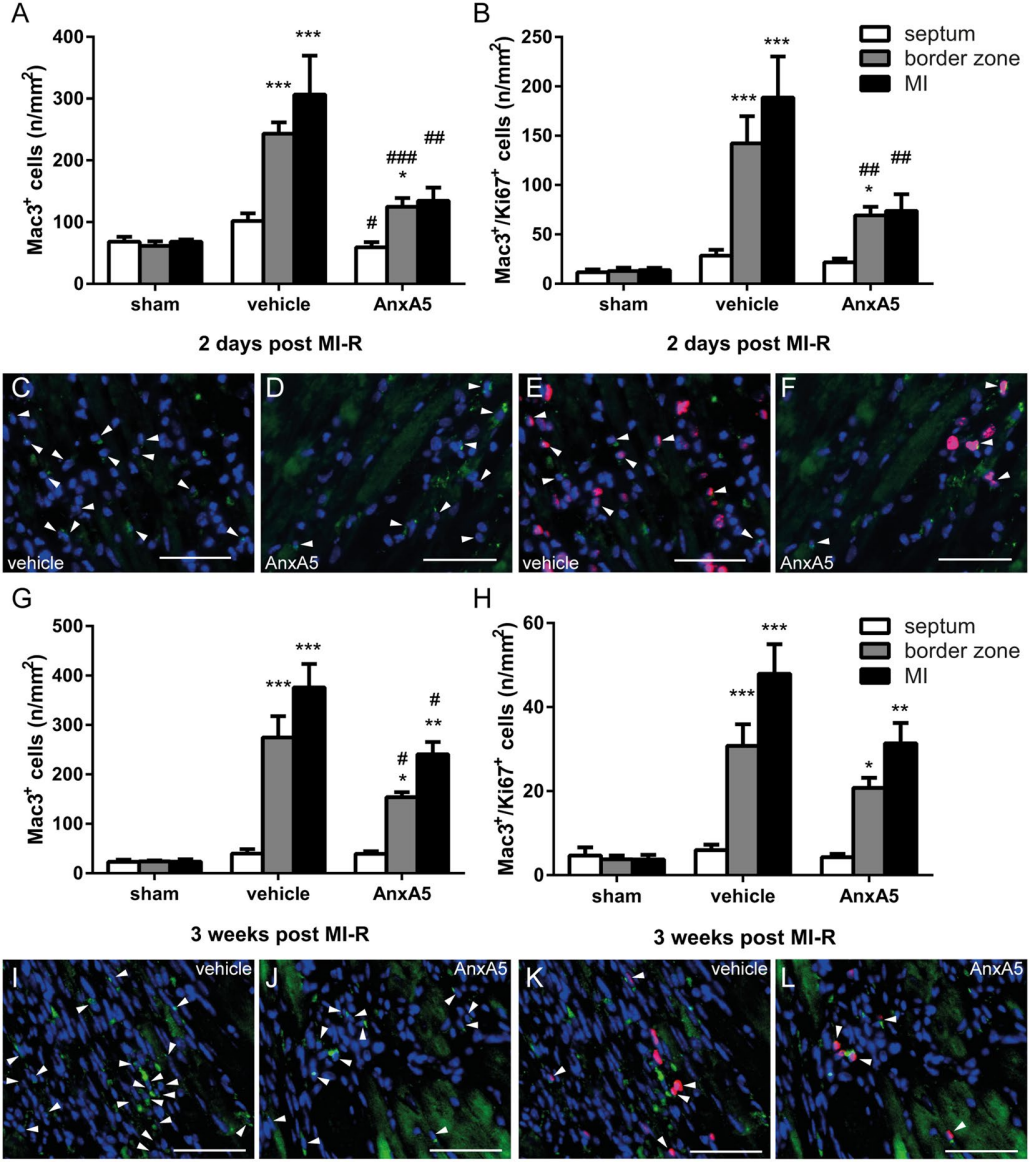
Local inflammatory response and macrophage proliferation. Quantification of the number of infiltrated macrophages showed an decreased number of infiltrated macrophages in the border zones and infarct area two days (n = 3–5) following MI-R in the AnxA5 group (**A**). Mac3/Ki67 double staining revealed a significant reduction in the number of proliferating macrophages in the border zones and infarct area after two days in the AnxA5 treated mice as compared to vehicle (n = 5–6; **B**). Three weeks after MI-R the number of infiltrated macrophages is significantly reduced in the border zones and infarct area upon AnxA5 treatment (**G**). No differences were observed regarding the number of proliferating macrophages three weeks after MI-R between AnxA5 and vehicle groups (**H**). Representative images of Mac3 staining (**C**,**D**,**I** and **J**) and Mac3/Ki67 double staining (**E**,**F**,**K** and **L**) of the infarct area. Nuclei are shown in blue, Mac3 staining in green and Ki67 in red, arrowheads indicate positive cells. Scale bar: 50 μm. Data are mean ± SEM. ^#^P,0.05, ^##^P < 0.01, ^###^P < 0.001 all vs. vehicle, *P < 0.05, **P < 0.01 and ***P < 0.001 all vs. sham.

Histological analysis two days after MI-R showed a significant increase in cardiac macrophages in both the vehicle (P < 0.001) and AnxA5 (P < 0.05) treated group when compared to sham mice, indicating an increase of the local inflammatory response following MI-R injury (Fig. 5A). Interestingly, the number of cardiac macrophages was reduced following AnxA5 treatment compared to vehicle in the infarct area (134.5 ± 21.3 vs. 306.4 ± 63.1 per mm^2^, P = 0.007), border zones (124.9 ± 14.1 vs. 243.0 ± 18.4 per mm^2^, P < 0.001) and interventricular septum (59.1 ± 8.4 vs. 101.9 ± 12.4 per mm^2^, P = 0.037). Taken together, this suggests, although increased compared to sham, AnxA5 treatment results in a reduction of the post-ischemic inflammatory response.

Three weeks after MI-R injury the number of cardiac macrophages is increased in both the vehicle (P < 0.001) and AnxA5 (P < 0.05) group when compared to the sham group, indicating the local inflammatory response is still increased following MI-R injury at this time point (Fig. 5G). Intriguingly, the number of cardiac macrophages was reduced following AnxA5 treatment compared to vehicle treated mice in the infarct area (240.6 ± 25.1 vs. 376.2 ± 47.3 per mm^2^, P = 0.029) and border zones (154.1 ± 9.9 vs. 274.6 ± 43.1 per mm^2^, P = 0.018), but not in the interventricular septum (39.3 ± 5.3 vs. 40.0 ± 8.7 per mm^2^, P = 1.000). Taken together, this means AnxA5 treatment not only reduces the early inflammatory response, but also the extended chronic inflammatory response.

### AnxA5 treatment reduces the number of proliferating macrophages

To further investigate the mechanism behind the observed reduced inflammatory response, serum CCL2 concentrations two and three weeks port MI-R injury were measured. No significant differences were observed between all groups regarding serum CCL2 concentrations at both time points (Supplementary Fig. S2).

Next, the number of proliferating macrophages (Mac3^+^/Ki67^+^ cells) was quantified. Two days post MI-R injury the number of proliferating macrophages is significantly reduced in the infarct area and border zones, but not in the interventricular septum in AnxA5 treated mice compared to vehicle treated mice (infarct area: 73.8 ± 17.0 vs. 188.6 ± 41.7, P = 0.009; border zones: 69.3 ± 8.6 vs. 142.2 ± 27.5, P = 0.008 and septum: 21.8 ± 3.7 vs. 28.5 ± 6.0 per mm^2^, P = 0.852; Fig. 5B). Compared to sham (infarct area: 13.9 ± 2.3, border zones: 12.9 ± 3.4 and septum: 11.5 ± 3.0 per mm^2^), vehicle treated mice show a significant increase in proliferating macrophages in the infarct area (P < 0.001) and border zones (P < 0.001) of the LV wall, while AnxA5 treated mice only show an increased number of proliferating macrophages in the border zones (P = 0.015).

Three weeks post MI-R injury no differences in proliferating macrophages could be observed between AnxA5 treated mice and vehicle treated mice (Fig. 5H). In the infarct area and border zones the number of proliferating macrophages was significantly increased in both the AnxA5 and vehicle group compared to the sham group (infarct area: AnxA5: 31.4 ± 4.9, P = 0.008,and vehicle: 47.9 ± 7.0, P < 0.001 both vs. sham: 3.7 ± 1.1 per mm^2^; border zones: AnxA5: 20.8 ± 2.4, P = 0.015 and vehicle: 30.8 ± 5.2, P < 0.001 both vs. sham: 3.8 ± 0.9 per mm^2^).

### AnxA5 stimulation reduces IL-6 production by macrophages *in vitro*

**Figure 6:**
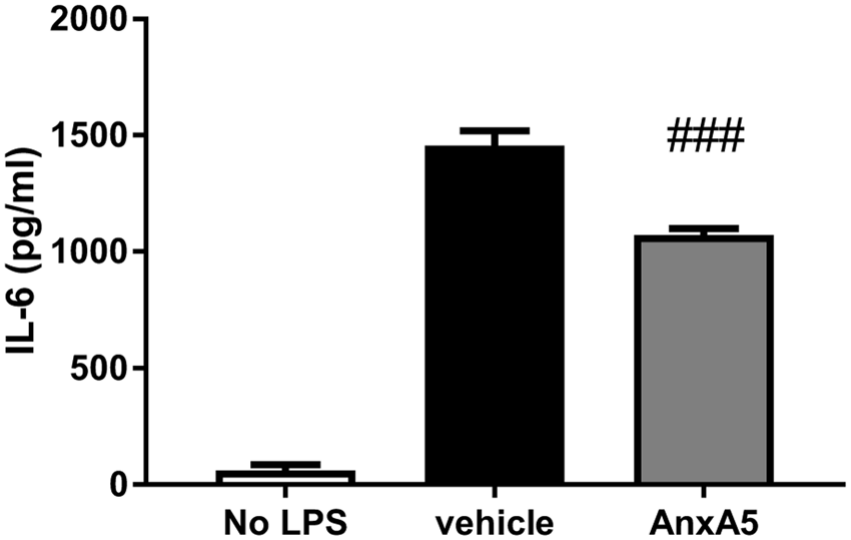
*In vitro* effect of AnxA5 stimulation on bone-marrow derived macrophages. IL-6 production was significantly reduced following AnxA5 stimulation (n = 5) compared to vehicle. Data are mean ± SEM. ^###^P < 0.001 vs. vehicle.

Finally, we investigated the effect of AnxA5 stimulation on cytokine production of bone-marrow derived macrophages (Fig. 6). AnxA5 stimulation reduces IL-6 production significantly compared to vehicle (1071 ± 28 vs. 1455 ± 65 pg/ml; P < 000.1).

## Discussion

This study shows a beneficial therapeutic effect of human recombinant AnxA5 administration following MI-R injury. By using hypercholesterolemic conditions and administering treatment directly after reperfusion results can be interpreted as clinically relevant. AnxA5 treatment resulted in accumulation of AnxA5 in the infarct area subsequently leading to a reduced infarct size, most likely mediated by attenuation of the inflammatory response. These results translated into limited LV dilation and improved cardiac function three weeks after MI-R. To our knowledge this is the first time that such a huge therapeutic effect (a 28.6% increase in EF) is shown after a follow-up of three weeks and treatment was started post-reperfusion. Although other groups have reported strong beneficial effects on EF of several compounds^22–24^, these studies started the therapeutic intervention before reperfusion, a setting that does not really mimics the situation seen in clinical treatment of patients. We found a striking increase in EF by 28.6% following post-reperfusion AnxA5 treatment.

Since infarct healing can be seen as a biphasic process with an inflammatory phase followed by a reparative and proliferative phase^25^, therapeutic effects are ideally analyzed after completion of both phases. Therefore we analyzed the effects of AnxA5 at two days and three weeks after MI-R. The fact that especially after three weeks AnxA5 treatment resulted in improved cardiac function and reduced infarct sizes, is very promising for the eventual translation of AnxA5 treatment to a clinical setting in which long term effects are desired.

Hypercholesterolemia is a primary risk factor for MI in human^26^ and it affects IS following MI-R injury in mice^27–29^ making the condition of value to implement in translational animal models. ApoE*3-Leiden mice develop hypercholesterolemia with subsequent atherosclerosis when fed a high-cholesterol diet, but not following a chow diet^30^. In our study we found a cardioprotective effect of AnxA5 treatment following MI-R in hypercholesterolemic ApoE*3-Leiden mice, although AnxA5 had no effects on plasma cholesterol levels. These mice mimic the clinical situation of most MI patients regarding hypercholesterolemia, and thereby adding more value to the found cardioprotective effect of AnxA5.

We demonstrated a reduced infarct size following MI-R injury as a result of AnxA5 treatment which was accompanied by preserved LV wall thickness of the infarcted area after three weeks. This might explain the improved cardiac function, since attenuation of post-ischemic LV remodeling is a prerequisite to improve LV function and finally attain prolonged survival^31^. In line with our results Hale *et al*. reported that therapeutic administration of Diannexin caused significantly smaller areas of no-reflow and obviously reduced infarct size within hours following severe myocardial ischemia in rabbits^21^. Diannexin, a 73 kDa recombinant dimer of the endogenous human Annexin A5 protein, has also been reported to have anti-thrombotic activities. Diannexin and annexin A5 bind PS with K(D) values of 0.6 and 5 nm, respectively, and both bind to the same subpopulation of PS-exposing platelets, thus inhibiting both the adverse effects of PS. Annexin A5 (35.7 kDa) is rapidly cleared from the circulation. In contrast to Annexin A5 which is rapidly cleared from the circulation, Diannexin, has an extended half-life, probably due to the higher molecular weight and the decreased renal clearance rate^21,32,33^. However, in this study we focus on the effects on natural occurring Annexin A5.

Molecular MR imaging with an annexin-labeled magnetofluorescent nanoparticle in combination with delayed-enhancement MRI has been shown to distinguish cardiomyocyte apoptosis from necrosis *in vivo* within 4 to 6 hours following MI-R injury^34^. Large areas of apoptotic but viable myocardium were revealed emphasizing its susceptibility to pharmacological intervention and salvation of apoptotic myocardium^34^. During the early phase of apoptosis in cardiomyocytes, endogenous AnxA5 is translocated and externalized^35,36^. Reversibility of this apoptotic process was demonstrated, as exogenous AnxA5 binds to externalized PS, thereby shielding of the “eat me” signal^18^. We suggest that treatment with AnxA5, like we performed in the current study, affects the exposed PS and thus attenuate the pro-apoptotic, but also the pro-inflammatory status of the myocardium. Up to at least 6 hours after a solitary MI-R insult AnxA5 binding to externalized PS resulted in restored sarcolemmal PS asymmetry after AnxA5 internalization^18^, which might be one of the mechanisms behind the observed decrease in IS after AnxA5 treatment.

Following AnxA5 treatment, the number of cardiac macrophages was dramatically decreased both two days and three weeks post MI-R compared to vehicle treatment. Macrophages play a dual role following MI-R injury, in the early phase after an infarction they help to clear the infarct area from cell debris and matrix components, which can act like damage associated molecular patterns, before they trigger the innate immune response^37^ as shown by Fig. 5 where an increase of cardiac macrophages can be found after MI-R in both AnxA5 and vehicle groups in comparison to the sham group. On the other hand, macrophages themselves can produce cytokines, which increase the inflammatory response^1,38^. This is one of the reasons for the disappointing results of present anti-inflammatory therapies in clinical trials, despite promising pre-clinical studies^39^. Therefore it seems plausible that rather than total abolishment of the inflammatory response, suppression of inflammation is beneficial following MI-R injury. This explains the observed striking beneficial results regarding IS and cardiac function following AnxA5 treatment, while the number of cardiac macrophages is increased compared to sham mice.

The cardiac macrophage population is maintained by both monocyte infiltration and local proliferation of macrophages. We found no differences in serum CCL2 concentrations, a critical player in monocyte recruitment to sites with tissue injury, following AnxA5 treatment. Therefore, we focused on the role of AnxA5 treatment on macrophage proliferation following MI-R injury. Interestingly, number of proliferating macrophages was significantly reduced two days post MI-R injury following AnxA5 treatment. However, the percentage proliferating macrophages was comparable in both the vehicle and AnxA5 group. Immediately following MI-R injury the resident macrophage population in the infarct area undergoes apoptosis and is replaced by mainly Ly6C^high^ monocytes, which differentiate into pro-inflammatory macrophages^40^. This population of pro-inflammatory macrophages is maintained by both recruitment of Ly6C^high^ monocytes as well as proliferation of the pro-inflammatory macrophages^41^. Since we did not observe differences in CCL2 concentrations and percentage proliferating macrophages was unaffected, the mechanism by which AnxA5 treatment reduces the number of cardiac macrophages is subject of future research.

We observed a decrease in IL-6 production by bone-marrow derived macrophages following AnxA5 stimulation. Recently, it has been shown that IL-6-deficient mice show reduced acute MI-R injury^42^. Furthermore, a correlation between IL-6 concentrations and myocardial damage was found in patients suffering from ischemic events^43^. This is in line with our results regarding IL-6 production by macrophages and the cardioprotective effect of AnxA5 *in vivo*. However, the exact mechanism by which AnxA5 regulates IL-6 production in this setting is subject for future research.

Radiolabeled AnxA5 is rapidly cleared from blood and accumulates in apoptotic tissue^20^. Previously, we have shown AnxA5 treatment leads to accumulation of AnxA5 at sites of injured vessel wall after systemic treatment, while AnxA5 is rapidly cleared from the blood^19^. In the current study, we show accumulation of AnxA5 in the infarct area following systemic AnxA5 treatment. The short half-life in blood, accumulation in the infarct area and cardioprotective effect suggests that AnxA5 is a safe and promising therapeutic agent.

Taken together, we showed a potential therapeutic role for human recombinant AnxA5 against MI-R injury in a clinical relevant setting. By suppression of the inflammatory response, AnxA5 attenuates long term adverse LV remodeling and improve cardiac function. A recent study by Ziegler *et al*., directed at the inhibition of inflammation using CD39 showed similar inhibitory effects on adverse post MI-R remodeling^44^. This underscores the new therapeutic potential of inhibiting inflammation in post MI-R remodeling.

## Methods

### Animals and diets

This study was performed in compliance with Dutch government guidelines and the Directive 2010/63/EU of the European Parliament. All animal experiments were approved by the Institutional Committee for Animal Welfare of the Leiden University Medical Center (LUMC). Transgenic female ApoE*3-Leiden mice^45^, backcrossed for more than 40 generations on a C57Bl/6 J background (bred in the animal facility of the LUMC), aged 8–10 weeks at the start of a dietary run-in period were used for this experiment. Mice were fed a semisynthetic Western-type diet supplemented with 0.4% cholesterol (AB Diets, Woerden, The Netherlands) 4 weeks prior to surgery, which was continued throughout the complete experiment. Mice were housed under standard conditions in conventional cages and received food and water ad libitum.

### Plasma lipid analysis

Plasma levels of total cholesterol (TC) and triglycerides (TG) were determined for randomization one week before surgery and at the end of the experiment. After a 4 hours fasting period, plasma was obtained via tail vein bleeding (~50 μL) and assayed for total cholesterol (TC) and triglycerides (TG) levels using commercially available enzymatic kits according to the manufacturer’s protocols (11489232 and 11488872, respectively; Roche Diagnostics, Mannheim, Germany).

### Surgical myocardial ischemia-reperfusion model and Annexin A5 administration

MI was induced by MI-R at day 0 in 12–14 weeks old female ApoE*3-Leiden mice as described previously^46^. Briefly, mice were pre-anesthetized with 5% isoflurane in a gas mixture of oxygen and room air and placed in a supine position on a heating pad (37 °C). After endotracheal intubation and ventilation (rate 160 breaths/min, stroke volume 190 μL; Harvard Apparatus, Holliston, MA, USA), mice were kept anesthetized with 1.5–2% isoflurane. Subsequently, a left thoracotomy was performed in the 4th intercostal space and the left anterior descending (LAD) coronary artery was ligated during 45 minutes using a 7–0 prolene suture knotted on a 2 mm section of a plastic tube followed by permanent reperfusion. Ischemia was confirmed by myocardial blanching. During this period muscle flaps were folded back and covered with a pre-warmed wet surgical mesh. Body temperature was kept constant between 35–37 °C. After 35 minutes of ischemia mice received an intraperitoneal injection of lidocain (6 mg/kg) to prevent cardiac arrhythmias caused by reperfusion. After 45 minutes of ischemia, permanent reperfusion was established. Subsequently, the thorax was closed in layers with 5–0 prolene suture and mice were allowed to recover. Analgesia was obtained with buprenorfine s.c. (0.1 mg/kg) pre-operative and 10–12 h post-operative. After surgery, animals were randomly grouped to receive daily administration of intraperitoneal injections with 1 mg/kg human recombinant annexin A5 (AnxA5; Athera Biotechnologies) in a volume of 200 μl, or NaCl 0.9% w/v (vehicle) as a control. Sham operated animals were operated similarly but without ligation of the LAD, and received intraperitoneal injections with NaCl 0.9% w/v. Injections were administered direct after surgery and between 12:00 p.m. and 2:00 p.m. the days thereafter. After two days or three weeks, mice were euthanized by bleeding and explantation of heart under general anesthesia with 1.5–2% isoflurane in a gas mixture of oxygen and room air. Hearts were quickly excised, immersion-fixated in 4% paraformaldehyde for 24 hours and embedded in paraffin. The heart and body weight were measured from all animals.

### Cardiac magnetic resonance imaging

Cardiac parameters were assessed two days and three weeks post MI-R using a 7-Tesla MRI (Bruker Biospin, Ettlingen, Germany) equipped with a combined gradient and shim coil, which is inserted into the magnet bore. Mice were pre-anesthetized as described above and kept anesthetized with 1.5–2% isoflurane. Respiratory rate was monitored by a respiration detection cushion, which was placed underneath the thorax and connected to a gating module to monitor respiratory rate (SA Instruments, Inc., Stony Brook, NY). Image reconstruction was performed using Bruker ParaVision 5.1 software.

### Infarct size

To determine infarct size, contrast enhanced MR imaging was performed after injection of a 150 µL bolus (0.5 mmol/ml) of gadolinium-DPTA (Gd-DPTA, Dotarem, Guerbet, the Netherlands) via the tail vein. A gradient echo sequence (FLASH) was used to acquire a set of 14 contiguous 0.7 mm contrast-enhanced slices in short-axis orientation covering the entire heart. Imaging parameters were: echo time of 1.9 ms, repetition time of 84.16 ms, field of view (33 mm^2^), and a matrix size of 192 × 256.

### Left ventricular function

Assessment of cardiac function was performed with a high-resolution 2D FLASH cine sequence to acquire a set of 9 contiguous 1 mm slices in short-axis orientation covering the entire heart. Imaging parameters were: echo time of 1.49 ms, repetition time of 5.16 ms, field of view (26 mm^2^), and a matrix size of 144 × 192.

### Image analysis

All MR image data was analyzed with the MASS for mice software package (MEDIS, Leiden, the Netherlands). The endocardial and epicardial borders were manually delineated and a reference point was positioned by an investigator blinded to treatment. Subsequently, the infarcted area of the LV, end-diastolic volume (EDV), end-systolic volume (ESV), ejection fraction (EF) were computed automatically.

### LV fibrous content and LV wall thickness

Paraffin-embedded hearts were cut into serial transverse sections of 5 μm along the entire long-axis of the LV and mounted on slides. To analyze collagen deposition as an indicator of the fibrotic area, every 50^th^ section of each heart was stained with Sirius Red. LV fibrous content, as a representation of IS, was determined by planimetric measurement of all sections and calculated as fibrotic area divided by the total LV wall surface area including the interventricular septum.

LV wall thickness was measured in five different sections centralized in the infarct area. Per section, wall thickness was analyzed at 3 places equally distributed in the infarcted area, both border zones, and 2 places of the interventricular septum. Measurements were performed perpendicular to the ventricular wall. Corresponding areas were used for measurements in the non-infarcted sham group. All measurements were performed by an observer blinded to the groups, using the ImageJ 1.47 v software program (NIH, USA).

### Immunohistochemistry

To evaluate AnxA5 accumulation in the infarct area paraffin section of the mid-infarct region of the heart were stained using antibodies against AnxA5 (anti-human annexin V, 3357–100, Biovision, Milpitas, CA, USA), while counterstaining was performed using haematoxylin.

For analysis of the cardiac inflammatory response paraffin sections of the mid-infarct region of the heart were stained using antibodies against macrophages (anti-Mac3, 550292; BD Pharmingen, San Diego, CA, USA) and to quantify the number of proliferating macrophages antibodies against proliferation marker Ki67 were used (anti-Ki67, ab16667; Abcam, Cambridge, UK). The number of macrophages and proliferating macrophages was expressed as a number per mm^2^ in the septum (2 areas), border zones (2 areas), and infarcted myocardium (3 areas).

### Bone-marrow derived macrophages

Bone-marrow derived cells were isolated from ApoE*3-Leiden mice and subjected to murine macrophage colony-stimulating factor (M-CSF) (20 ng/μl; Miltenyi Biotec) to stimulate differentiation into macrophages.

Macrophages were stimulated by exposure to 8% heat-inactivated FCS in the presence and absence of AnxA5 (2 µM). The cells were incubated overnight at 37 °C in 5% CO2 atmosphere. Next, bone marrow derived macrophage were stimulated with or without LPS (10 ng/ml) for six hours and the supernatants were collected. Subsequently, IL-6 production by these bone marrow-derived macrophages was analyzed by ELISA.

### ELISA

To study the effects of AnxA5 on systemic inflammation, an ELISA kit (Cat. No. 555260, BD Biosciences, San Diego, CA, USA) for cytokine concentration of chemokine (C-C motif) ligand 2 (CCL2) was used. Furthermore, the IL-6 production by bone-marrow derived macrophages *in vitro* was measured using an ELISA kit (Cat. No. 555240 (IL-6), BD Biosciences, San Diego, CA, USA), according to the manufacturer’s instructions.

### Statistical analysis

Values were expressed as mean ± SEM. Comparisons of parameters between the sham, AnxA5, and vehicle groups were made using 1-way analysis of variance (ANOVA) with Bonferroni’s correction or 2-way ANOVA with repeated measures and Bonferroni’s post-test in case of multiple time points. Comparisons between AnxA5 and vehicle were made using (un)paired t-tests. A value of P < 0.05 was considered to represent a significant difference. Statistical procedures were performed using IBM SPSS 23.0.0 (SPSS Inc – IBM, Armonk, NY, USA) and GraphPad Prism 6.02 (GraphPad Software Inc, La Jolla, CA, USA).

## Electronic supplementary material

Supplementary information
