# Supplementary material for: Annexin A5 reduces infarct size and improves cardiac function after myocardial ischemia-reperfusion injury by suppression of the cardiac inflammatory response
Source: Sci Rep. 2018 Apr 30;8:6753. doi: 10.1038/s41598-018-25143-y (PMC5928225; doi:10.1038/s41598-018-25143-y)
Supplement: Supplementary file 1 — Supplementary information [file 41598_2018_25143_MOESM1_ESM.doc]

**Supplementary information**

**Annexin A5 reduces infarct size and improves cardiac function after myocardial ischemia-reperfusion by suppression of the cardiac inflammatory response**

Rob C. M. de Jong^1,2^*, Niek J. Pluijmert^3^*, Margreet R. de Vries^1,2^, Knut Pettersson^4^, Douwe E. Atsma^3^, J. Wouter Jukema^1,3^, Paul H. A. Quax^1,2^

*Authors contributed equally to this work

**
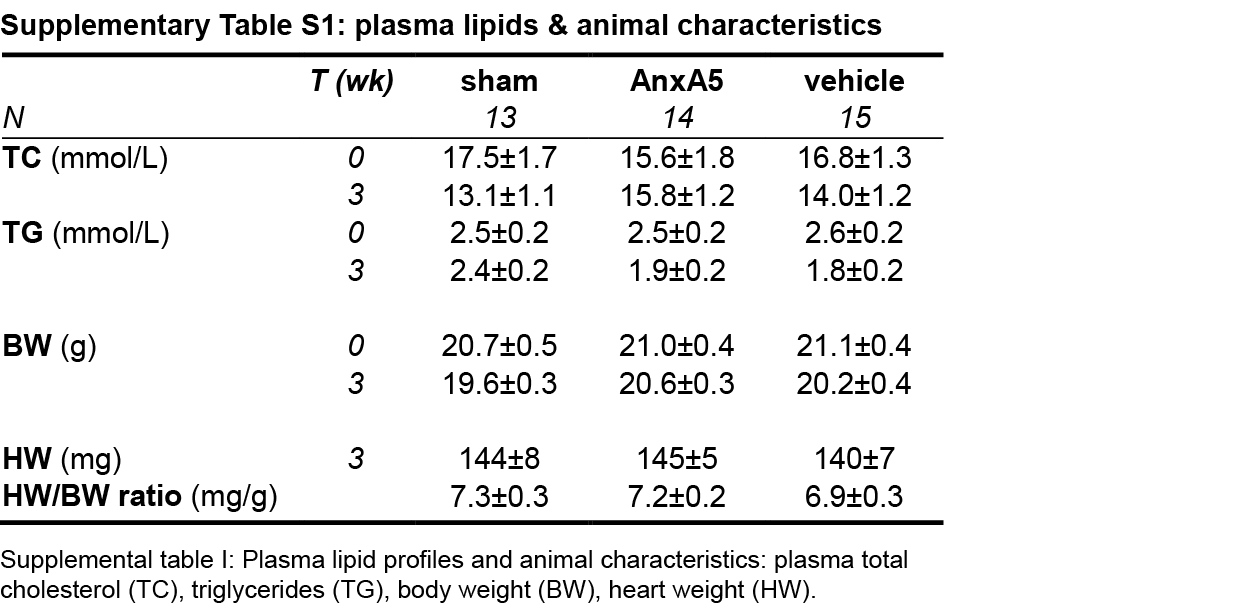
**

**Supplementary figures + legend**

**
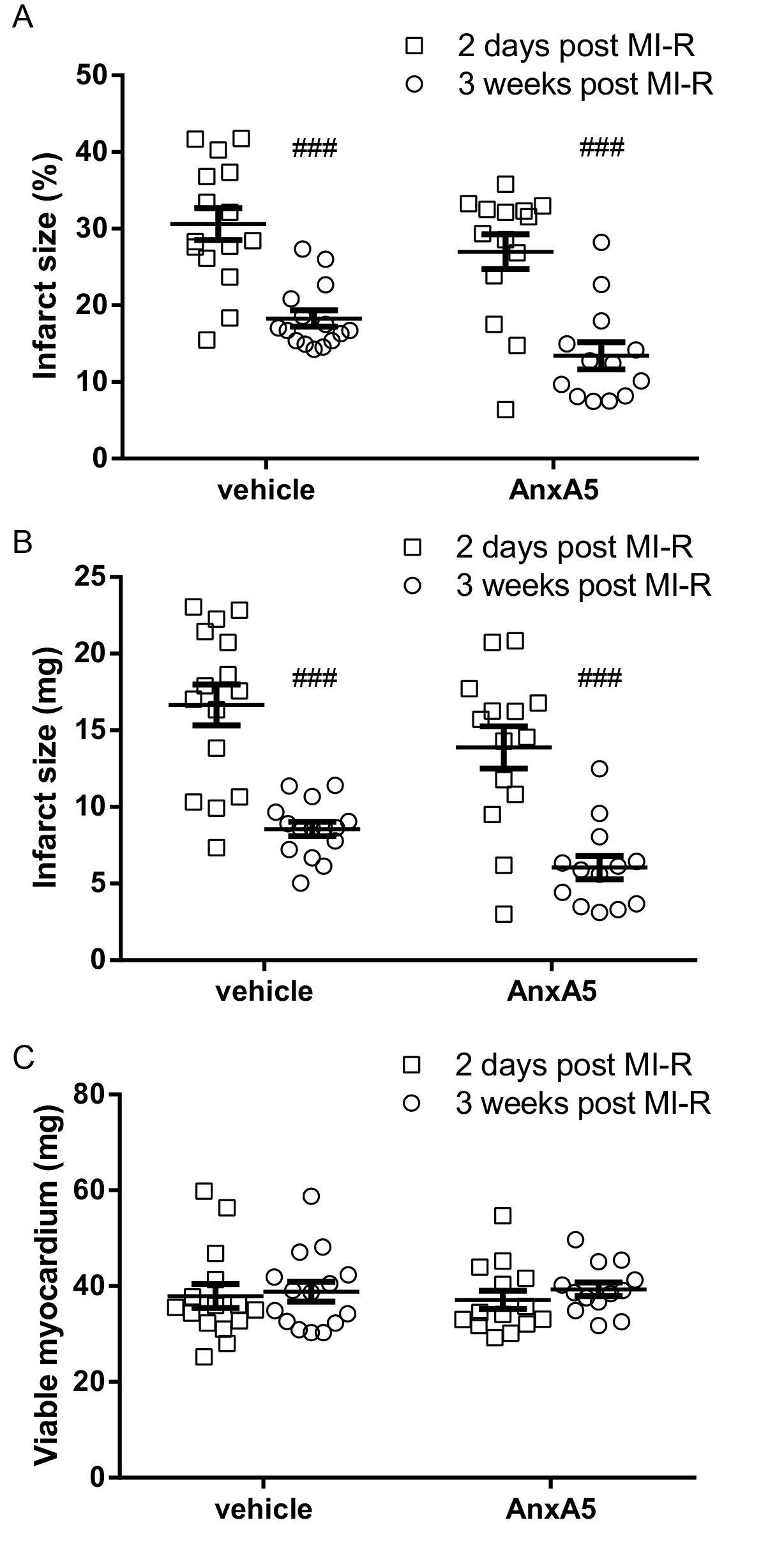
**

**Supplementary figure S1: Absolute numbers contrast-enhanced MR imaging.** Three weeks after MI-R IS was significantly smaller in both the vehicle as the AnxA5 group compared to two days after MI-R (A and B). However, viable myocardium remained unchanged in both groups (D). Data are means ± SEM. ###P<0.01 vs. 2 days post MI-R.

**
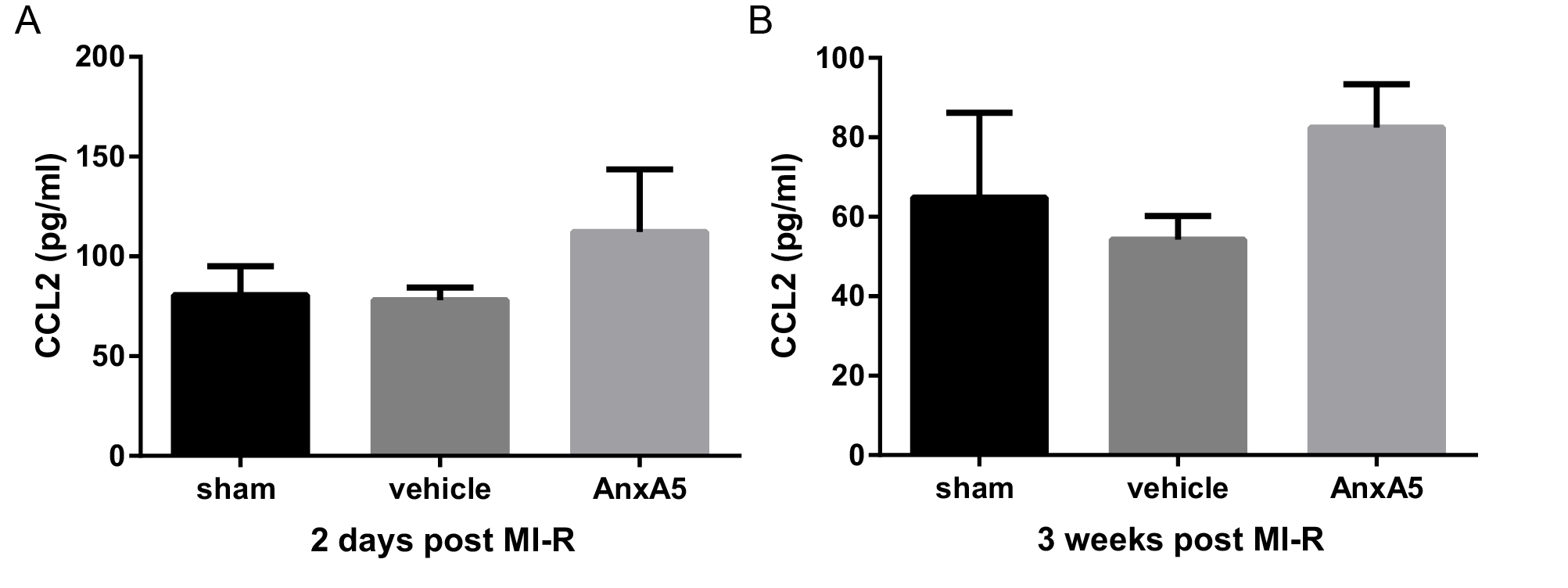
Supplementary figure S2: Serum CCL2 concentrations.** Both two days (A) and three weeks (B) post MI-R injury no differences could be observed in serum CCL2 concentrations between all groups.

**
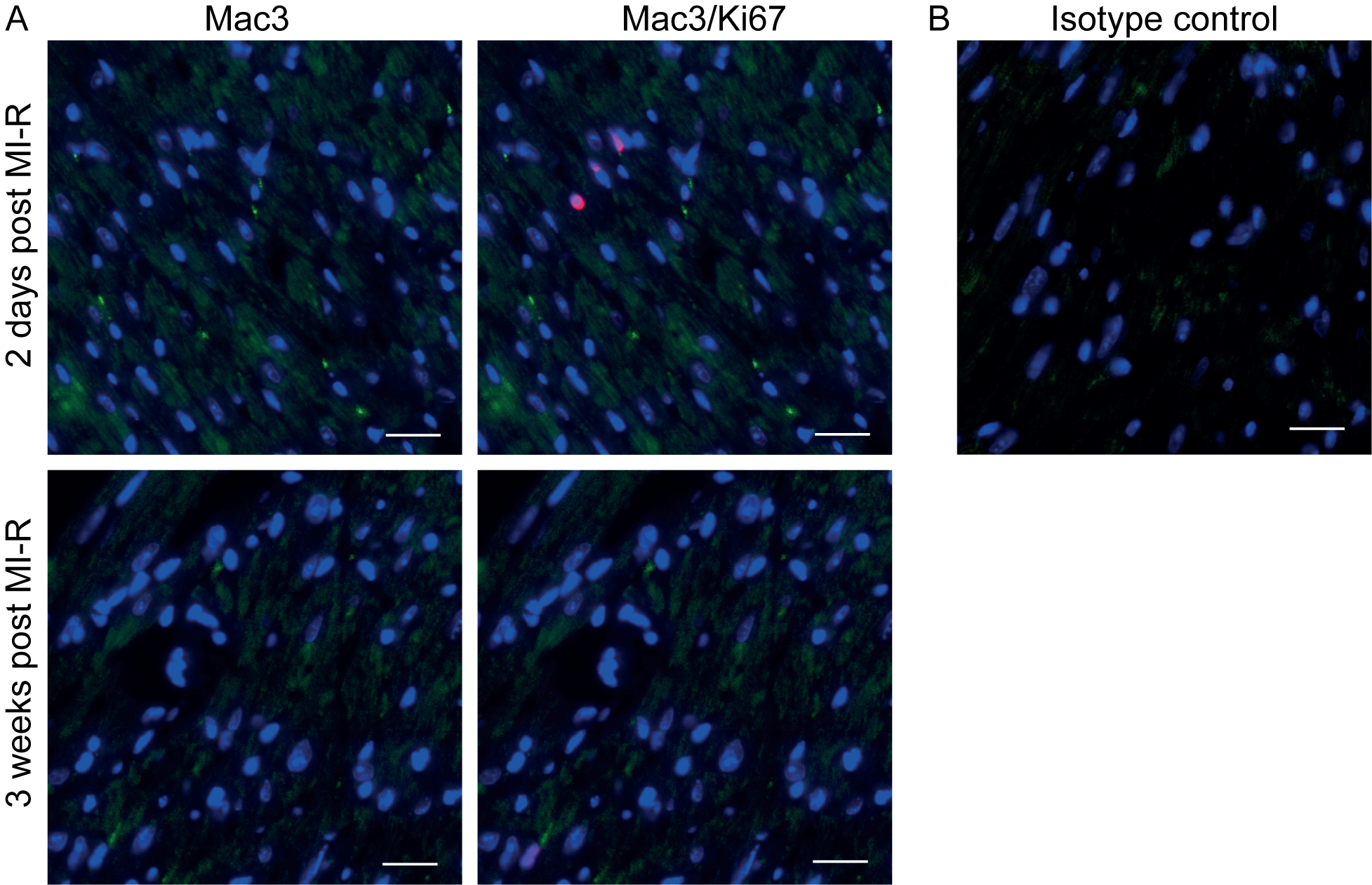
**

**Supplemental figure S3: Representative images of Mac/Ki67 staining.** Representative images of Mac3 staining (left) and Mac3/Ki67 double staining (right) of the sham group infarct area (A). Nuclei are shown in blue, Mac3 staining in green and Ki67 in red, arrowheads indicate positive cells. Representative image of isotype control (B). Scale bar: 20 µm.
